# A Deep Learning-Based Latent Trait Model for Forced-Choice Personality Assessment

**DOI:** 10.3390/bs16071140

**Published:** 2026-07-07

**Authors:** Xiaoyu Li, Jin Wu, Yupei Ren, Shaoyang Guo, Zhongquan Li, Chanjin Zheng

**Affiliations:** 1 School of Education and Intelligent Education Research Center, Yangzhou University, Yangzhou 225009, China; syguo1992@yzu.edu.cn; 2 Shanghai Institute of Artificial Intelligence for Education, East China Normal University, Shanghai 200062, China; 52275901018@stu.ecnu.edu.cn (J.W.); ypren@stu.ecnu.edu.cn (Y.R.); 3 Department of Psychology, Nanjing University, Nanjing 210023, China; zqli@nju.edu.cn

**Keywords:** latent trait model, forced-choice test, deep learning, intelligent personality assessment

## Abstract

In the era of intelligent assessment, psychometric tests are becoming increasingly important for personnel selection, career development, and mental health assessment. Forced-choice tests are common in personality assessments because they require participants to select from closely related options, lowering the risk of response distortion. However, traditional latent trait models for forced-choice tests suffer from severe computational bottlenecks in high-dimensional settings. Furthermore, existing deep learning-based cognitive diagnosis models are primarily designed for independent items in educational scenarios (predicated on absolute scoring), making them structurally maladapted to the ipsative data (relative preference comparisons) generated by forced-choice tests. To address these challenges, this study presents a deep learning-based Forced-Choice Neural Latent Trait (FCNLT) Model that overcomes the limitations of traditional models and is applicable to the three most common item block types found in forced-choice tests. To account for the unidimensionality of items, participants’ latent trait levels and item characteristics are represented as interpretable latent embeddings. FCNLT mines these features through nonlinear mapping and introduces a weighted BPR-based ranking loss to natively align with the relative-scoring nature of forced-choice data. Additionally, the monotonicity assumption is utilized to improve the interpretability of the trait estimates. The FCNLT’s effectiveness is validated by experiments on real-world and simulated datasets that show its accuracy, interpretability, and robustness.

## 1. Introduction

In the age of intelligence, psychometric assessments have become increasingly essential. They not only equip individuals with tools for self-awareness but also provide organizations with a scientific foundation for personnel selection (Bateson et al., 2014; Robertson & Smith, 2001), career development (Savickas & Hartung, 1996), and mental health assessment (Mueser et al., 2001). Psychometric tests are typically divided into cognitive and noncognitive categories. Cognitive tests assess abilities such as logical reasoning and typically use standardized answers, with higher scores indicating greater ability. Intelligent education leverages cognitive testing in various ways, including knowledge tracing (Abdelrahman et al., 2023; Piech et al., 2015), cognitive diagnosis (Q. Liu et al., 2018; F. Wang et al., 2022), and personalized learning (Shemshack & Spector, 2020). Non-cognitive tests, on the other hand, are critical for understanding personality traits, values, and attitudes. They are widely used in clinical diagnosis, career planning, and personnel decisions. Research consistently shows that personality assessments have strong predictive validity for job performance (Brown & Bartram, 2011; Hurtz & Donovan, 2000; Sitser et al., 2013). Likert scales are used in many non-cognitive tests, including the Minnesota Multiphasic Personality Inventory (MMPI) (Hathaway & McKinley, 1951) and the Cattell 16 Personality Factor Inventory (16PF) (Cattell, 1956). However, in high-stakes situations, participants may be influenced by social desirability, causing them to select responses that do not accurately reflect their true traits, jeopardizing the fairness and discriminability of the outcomes.

**Figure 1:**
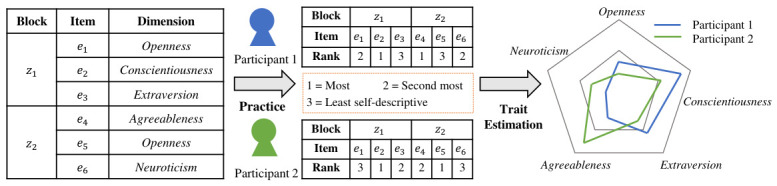
A toy example of latent trait estimation in forced-choice assessment.

To address this issue, researchers have developed forced-choice question formats to effectively mitigate faking and social desirability bias in high-stakes assessments (M. Cao & Drasgow, 2019; Jackson et al., 2000; Saville & Willson, 1991; Wetzel et al., 2021). These tests require participants to choose between two or more options of comparable social desirability, which reduces the possibility of response distortion. The forced-choice task’s format is determined by the item display mode, which can be one of three: PICK, RANK, or MOLE. As shown in Table 1, the PICK format requires participants to choose the statement in an item block that is most descriptive of themselves; the RANK format requires participants to rank the statements within a block in order of decreasing self-descriptiveness; and the MOLE format requires participants to choose both the most and least descriptive statements within the block. Figure 1 shows the schematic of the latent trait estimation process for the RANK item block type, where participants evaluate the endorsement tendency of the provided descriptions. Based on the participants’ responses to the items, an appropriate latent trait model was selected to estimate their latent trait levels.

Traditional multidimensional forced-choice (MFC) tests are scored using either conventional scoring techniques or item response theory (IRT). IRT models for forced-choice tests have been developed to determine the relationship between responses and latent traits, generate normative latent trait scores, and allow for inter-individual score comparisons. Common models include Thurstonian Item Response Theory (TIRT) (Brown & Maydeu-Olivares, 2011) and Multi-Unidimensional Pairwise Preferences (MUPP) (Stark et al., 2005). For parameter estimation, these models often use EM algorithm versions (e.g., iStEM, SAEM, and MHRM) or MCMC. However, as the number of responders and items grows, so does the number of model parameters, resulting in a significant rise in both memory consumption and computing time. For instance, Zheng et al. (2024) reported that when the number of dimensions increased from 12 to 24 and the number of item blocks from 120 to 480, the MCMC estimation time rose from 11.500 h to 47.954 h, and the iStEM estimation time rose from 0.783 h to 2.817 h. Such computational overhead highlights the need for more efficient computational approaches to high-dimensional forced-choice data.

The rapid advancement of data science and deep learning technologies has made deep learning-based cognitive diagnostic models^1^ (CDMs) a popular research topic. Leveraging their strong feature extraction capabilities, these models are able to extract patterns from sparse data and capture complex interactions among features, demonstrating significant advantages in educational test data (L. Gao et al., 2022; S. Liu et al., 2024; Qi et al., 2023; F. Wang et al., 2020). However, existing deep learning-based cognitive diagnostic models are primarily intended for educational testing scenarios and cannot be used directly for forced-choice tests. The main differences are evident in the following three aspects: **(1) Mismatch in data format.** Traditional educational tests and Likert scales use absolute scoring (e.g., item scores), whereas forced-choice tests ask responders to make relative comparisons between items within an item block. Existing models (e.g., NCDM (F. Wang et al., 2020), KaNCD (F. Wang et al., 2022), and CDND (Y. Zhang et al., 2025)) rely on independent scores as input and cannot directly handle intra-block comparisons. Some models can be improved by introducing ranking algorithms, with the original model serving as a component for estimating endorsement probability and forecasting item preference. **(2) Mechanisms for mapping items to dimensions are inconsistent.** Both educational and forced-choice tests are intended to measure multiple dimensions, but the mapping of items to dimensions differs. In educational tests, an item may include multiple knowledge components, necessitating the use of models that account for the entire knowledge component space. Forced-choice tests, on the other hand, use a strictly unidimensional design for each item, with mutually exclusive dimensions within the block. This causes significant parameter redundancy in existing models (i.e., only a portion of the parameters are used in training for each item), lowering training efficiency. **(3) Differences in data characteristics.** Models in educational contexts frequently use specific design elements such as guessing and slipping parameters (de la Torre, 2009; S. Wang et al., 2024) or graph structures based on response correctness (S. Liu et al., 2024). However, forced-choice test data lacks these characteristics, so existing models cannot be directly transferred to the forced-choice domain. As a result, developing deep learning models specifically designed for forced-choice tests has become a pressing issue that must be addressed.

To address these issues, this study proposes the Forced-Choice Neural Latent Trait (FCNLT) model for evaluating forced-choice personality tests. First, participant and item embeddings are created using one-hot encoding. Due to the unidimensional nature of forced-choice items, participant and item features are created and mapped into a high-dimensional space. A two-layer non-negative fully connected network, with a monotonicity assumption, is used to simulate the complex interactions underlying participants’ response processes. Second, the Bayesian Personalized Ranking (BPR) loss function is tailored to the characteristics of forced-choice tests, maximizing differentiation of self-descriptiveness among items within a block. Finally, experiments are carried out using three real-world datasets and one simulated dataset. Two evaluation metrics are proposed: Pairwise Rank Accuracy (PRA) and Listwise Rank Accuracy (LRA), which evaluate the model’s ranking prediction performance in terms of item pairs and entire item blocks, respectively.

The main contributions of this study are as follows:

- The FCNLT model was proposed specifically for forced-choice personality tests, with the goal of overcoming the limitations of traditional psychometric models in this domain.
- The FCNLT model supports the three most common forced-choice formats (PICK, RANK, and MOLE) and includes an updated BPR loss function to better match the characteristics of forced-choice datasets.
- The PRA and LRA metrics were used to assess the model’s ranking predictive power, and thorough tests were carried out on both real-world and simulated datasets to confirm the proposed model’s efficacy, interpretability, and robustness.

## 2. Related Work

This section reviews three lines of work relevant to this study. Section 2.1 introduces the types and models of forced-choice tests. Section 2.2 traces the development of deep learning-based cognitive diagnostic models. Section 2.3 discusses pairwise and listwise ranking algorithms. Together, these reviews provide the technical foundation for the proposed approach.

### 2.1. Types and Models of Forced-Choice Tests

Forced-choice personality tests have two scoring approaches:traditional scoring methods and latent trait models. This section introduces both approaches.

#### 2.1.1. Traditional Scoring Methods

Traditional scoring methods provide 1 point to the most self-descriptive or ranked highest item within an item block, −1 point to the item regarded as least self-descriptive or ranked lowest, and 0 points to unselected or intermediately ranked items. The overall score for each dimension is calculated by adding the scores of items within that dimension. Although this method is straightforward and easy to apply, it yields ipsative data; because the sum of item scores within each item block equals zero, the overall score across the entire test equals zero. As a result, scores across dimensions become interdependent, with high scores on some dimensions implying low scores on others. It is impossible to have all dimensions simultaneously high or low (Brown & Maydeu-Olivares, 2011). The interdependence of ipsative data violates the fundamental concept of independence of error variances in classical test theory, which has implications for the statistical analysis and interpretation of forced-choice test scores (Baron, 1996). The reliability of ipsative data is determined by the number of dimensions and their correlations. The issues connected with ipsative data are more severe in tests measuring fewer dimensions (Clemans, 1966). Ipsative data become unreliable when the number of dimensions is less than 10 or the inter-dimension correlations exceed 0.3 (Bartram, 1996). When there are more than 30 dimensions and low inter-dimension correlations, ipsative data become reliable enough to be regarded as normative data and used for inter-individual comparisons (Saville & Willson, 1991).

#### 2.1.2. Latent Trait Models

To address the issue of ipsative data in traditional scoring methods, researchers created latent trait models for forced-choice tests. These models aim to build relationships between observed responses and latent traits, which leads to latent trait scores with normative properties and score comparability among individuals.

These models can be categorized into two kinds based on the predicted item response patterns: dominance models and unfolding models. Dominance models hold that the higher an individual’s level on a trait, the higher the probability of endorsing a relevant item; common examples are the Rasch model and the two-parameter logistic (2PL) model. Unfolding models, in contrast, assume that the probability of endorsement is directly related to the item’s proximity to the individual’s trait level (Drasgow et al., 2010). One such example is the Generalized Graded Unfolding Model (GGUM) (Roberts et al., 2000). In the context of forced-choice tests, the commonly used decision theories fall into two main categories. The first is Thurstone’s Law of Comparative Judgment (Thurstone, 1927), and the second is Luce’s choice axiom (Luce, 1959) along with the Bradley–Terry model (Bradley & Terry, 1952), the latter being a special case of the former (Brown, 2016).

Commonly used latent trait models for forced-choice tests include the TIRT model (Brown & Maydeu-Olivares, 2011), which is based on Thurstone’s law of comparative judgment and is suitable for dominance response items as well as for forced-choice formats such as PICK-2, RANK, and MOLE. Another major framework is the MUPP approach and its derivatives, initially developed for the PICK-2 format (Stark et al., 2005)—for instance, MUPP-GGUM, which falls under unfolding response models. Subsequent work extended the MUPP framework to dominance response models, yielding MUPP-2PL (Morillo et al., 2016), which also applies to the PICK-2 format. Building on Luce’s choice axiom, MUPP-RANK (Hontangas et al., 2015) further broadens the applicability of the MUPP family to additional forced-choice formats, including PICK, RANK, and MOLE. More recently, two new dominance models have been proposed. First, the G-DINA model has been adapted for forced-choice blocks, providing a flexible framework for diagnostic classification with unidimensional statement pairs under the PICK format (Nájera et al., 2025). Second, the multidimensional generalized partial preference model (MGPPM), which also falls within the category of dominance models, applies to the PICK, RANK, and MOLE formats and provides a statistically more parsimonious alternative to rank-based models (Furr & Fu, 2025). In addition to these general frameworks, several specialized models have been developed. These include the RIM model (W.-C. Wang et al., 2017) for obtaining intra-individual comparative scores, and the BRB-IRT model (Lee & Smith, 2020), which accounts for random block effects. Table 2 summarizes the most widely used models.

### 2.2. Deep Learning-Based Cognitive Diagnostic Models

With the increasing diversity of test formats and the rise of data-driven methods, researchers have begun to introduce deep learning into the field of cognitive diagnosis, leading to a significant paradigm shift in the area. Early explorations, represented by the Neural Cognitive Diagnosis Model (NCDM) (F. Wang et al., 2020), replaced traditional mathematical functions with multi-layer neural networks to directly model the nonlinear interactions among student proficiency, knowledge difficulty, and item discrimination. This approach significantly improved model fit while preserving the interpretability essential to psychometric measurement. Subsequently, deep learning architectures were further extended. On one hand, models such as KaNCD (F. Wang et al., 2022), KSCD (Ma et al., 2022), and ICD (Qi et al., 2023) deepened the modeling of interactions among knowledge concepts. On the other hand, models such as RCD (W. Gao et al., 2021) and ICDM (S. Liu et al., 2024) introduced graph neural networks to capture the topological structure among students, items, and knowledge concepts. Additionally, subsequent studies have extensively explored the potential of deep learning in handling complex measurement scenarios, including multimodal data (Song et al., 2023), long-tail problems (Ma et al., 2024; S. Wang et al., 2023; Yao et al., 2023), cold-start issues (W. Gao et al., 2024), contextual, affective features (S. Wang et al., 2024; Zhou et al., 2021), and graph-based cognitive diagnosis (G. Zhang et al., 2026).

As test formats have diversified, deep learning-based cognitive diagnosis models have also evolved from binary to polytomous scoring data. To accommodate polytomous data such as subjective items or Likert scales, FuzzyCDF (Q. Liu et al., 2018) developed a fuzzy set that transforms polytomous scores into continuous values from 0 to 1. This dimensionality reduction method was later adopted by models such as SPP-NCD (Ma et al., 2023), deepCDF (L. Gao et al., 2022), and QRCDM (Yang et al., 2022). However, this score mapping method only yields approximate estimates of participant ability, overlooks item parameter differences across score levels, and struggles to accurately predict specific scores. To address this issue, PCDF (Li et al., 2025) uses graded response model theory to create a cognitive diagnosis framework based on data re-encoding, allowing polytomous score data to be more easily interpreted.

In summary, although existing deep learning-based CDMs have made notable progress in handling both binary and polytomous scoring data, a review of the literature reveals that the evolutionary trajectory of these models remains deeply embedded in the paradigm of traditional educational testing. Specifically, most existing deep architectures are built upon the assumptions of local independence and absolute scoring mechanisms, and their network designs often heavily rely on characteristics specific to educational data, such as parameter fitting for guessing and slipping behaviors (S. Wang et al., 2024) or graph structures constructed based on response correctness (S. Liu et al., 2024). In contrast, forced-choice tests, which are widely used in the assessment of personality and non-cognitive traits, require participants to make relative preference comparisons within item blocks, thereby generating ipsative data and adhering to a strictly unidimensional and mutually exclusive item design within each block. At present, the deep learning cognitive diagnosis literature has not built architectures that follow the idea of relative comparison. Directly transferring the aforementioned complex full-space networks designed for educational contexts to forced-choice tests would inevitably result in substantial parameter redundancy and a fundamental mismatch in mechanisms. Therefore, bridging the gap in data types and underlying mechanisms to develop neural network architectures suitable for forced-choice tests represents not only an inevitable requirement for the evolution of modern measurement models but also a critical theoretical gap that urgently needs to be addressed in the current field of cognitive diagnosis.

### 2.3. Ranking Algorithm

Since forced-choice tests require participants to make relative comparisons between multiple items in the same item block, their primary data structure necessarily includes ranking information. To deal with such data, this study incorporates the concept of ranking algorithms into the design of latent trait models. Existing ranking algorithms are divided into two types based on the granularity of optimization: pairwise ranking and listwise ranking. The following sections introduce these two types of algorithms and their applications in the field of educational measurement.

#### 2.3.1. Pairwise Ranking Methods

Pairwise ranking methods were initially developed for recommendation systems, where models are optimized by comparing user preferences for items. BPR (Rendle et al., 2009) is a representative approach that aims to maximize the difference in scores between preferred and non-preferred items, therefore enhancing ranking performance. The advantage of BPR lies in its simplicity and efficiency, as it can be optimized simply utilizing implicit feedback data. RankNet (Burges et al., 2005) optimizes models by minimizing the disparity between anticipated and true ranks. LambdaRank (Burges et al., 2007) improves ranking performance by directly improving information retrieval evaluation measures like NDCG.

In the field of education, IRR (Tong et al., 2021) constructs positive and negative response pairs for the same item across different participants to reinforce the monotonicity assumption, thereby enhancing model predictive performance. To solve the issue of long-tail data distributions, researchers frequently combine unattempted data with existing response data to produce positive and negative pairs, then optimize using the BPR loss function. For example, the EIRS (Yao et al., 2023) and CMES (Ma et al., 2024) models use this strategy for effective ranking optimization. However, forced-choice tests construct pairs by comparing different items within the same participant, resulting in optimization objectives that differ from those of the aforementioned models. Consequently, these models cannot be directly applied to forced-choice testing contexts.

#### 2.3.2. Listwise Ranking Methods

Listwise ranking methods optimize the ranking structure of an entire list, allowing for a more comprehensive capture of global ranking information. Classic approaches include ListNet (Z. Cao et al., 2007), which optimizes the model by minimizing the KL divergence between the predicted ranking distribution and the target ranking distribution, and ListMLE (Xia et al., 2008), which employs maximum likelihood estimation to directly optimize the probability distribution over ranked lists. AdaRank (Xu & Li, 2007) iteratively modifies model parameters to maximize ranking performance. LambdaMART (Burges, 2010) combines the strengths of gradient boosted trees with LambdaRank, significantly improving ranking effectiveness.

## 3. Methods

### 3.1. Research Background

#### 3.1.1. Problem Definition

Here is a formal definition of a forced-choice test. Suppose there are *N* participants, *M* Items, *K* dimensions and *L* item block in a test, denoted $S=\{s_{1},s_{2},\ldots ,s_{N}\}$, $E=\{e_{1},e_{2},\ldots ,e_{M}\}$, $C=\{c_{1},c_{2},\ldots ,c_{K}\}$, and $Z=\{z_{1},z_{2},\ldots ,z_{L}\}$, where each item block $z_{l}$ contains *t* items, i.e., $z_{l}\subseteq E$. During the answering process, each participant $s_{n}$ ranks the items in item block $z_{l}$ based on how well the descriptions fit them. The participant’s response log is denoted as $R=(s_{n},z_{l},r_{n,l})$, where $r_{n,l}$ represents the ranking result of participant $s_{n}$ on item block $z_{l}$.

Depending on the item format, the specific form of $r_{n,l}$ is as follows:

- **PICK:** Participants select the most conforming item from item block $z_{l}$. This item is assigned a value of *t*, while all other items receive a value of 1.
- **RANK:** Participants are required to perform a complete ranking of all items in block $z_{l}$, namely $\left\{e_{(l-1)*t+1},e_{(l-1)*t+2},\ldots ,e_{l*t}\right\}$. The most self-descriptive item is assigned a value of *t*, the second most self-descriptive item is assigned t−1, and so forth, with the least self-descriptive item assigned a value of 1.
- **MOLE:** Participants choose one most conforming and one least conforming item from item block $z_{l}$. The most conforming item is assigned a value of 3, the least conforming a value of 1, and all other items receive a value of 2.

Additionally, the test has a Q-matrix $Q={\left\{Q_{mk}\right\}}_{M\times K}$, where $Q_{mk}=1$ if item $e_{m}$ relates to dimension $c_{k}$ and $Q_{mk}=0$ otherwise.

**Problem Definition:** Given the participants’ response logs *R* and the Q-matrix *Q*, our goal is to predict the ordering of items within an item block as closely as possible to the participants’ true ranking results.

#### 3.1.2. Monotonicity Assumption

The monotonicity assumption (Rosenbaum, 1984) is a fundamental principle in psychometric modeling, especially applied to dominance models. It ensures logical consistency between the assessment of latent traits and observed behavioral performance. This assumption holds that for higher participant trait levels on a given cognitive attribute, the probability of endorsing the corresponding statement should exhibit a non-decreasing trend (i.e., remain at least unchanged or increase). In other words, as participants’ overall ability improves or their mastery of certain attributes strengthens, their probability of endorsement tendency should not decrease. This property is critical to the measurement validity of a model, as violations of the monotonicity assumption may lead to high-trait-level individuals being misclassified as low-trait-level or may obscure genuine trait profiles.

Existing cognitive diagnosis methods typically satisfy the monotonicity assumption by embedding monotonicity constraints into the interaction function. Earlier research on NCDM (F. Wang et al., 2020) required non-negative weights for completely connected layers to ensure monotonicity. This strategy has since been widely used in subsequent studies (F. Wang et al., 2022; S. Wang et al., 2024; Y. Zhang et al., 2025). Given its effectiveness and generalizability, the present study also uses this approach to ensure the trait estimates are acceptable and interpretable.

### 3.2. Design of Forced-Choice Neural Latent Trait Model

Most existing deep learning-based cognitive diagnostic models follow a general one-hot encoding, feature construction, and interaction function pipeline, with variations in specific details and innovations. FCNLT also adopts this general paradigm and retains the IRT-inspired interaction function. On this basis, two key modifications are introduced for forced-choice tests. First, participant representations are restricted to the single dimension relevant to each item, avoiding interference across trait dimensions. Second, a weighted BPR loss function is introduced to capture the relative preference strength between items.

**Figure 2:**
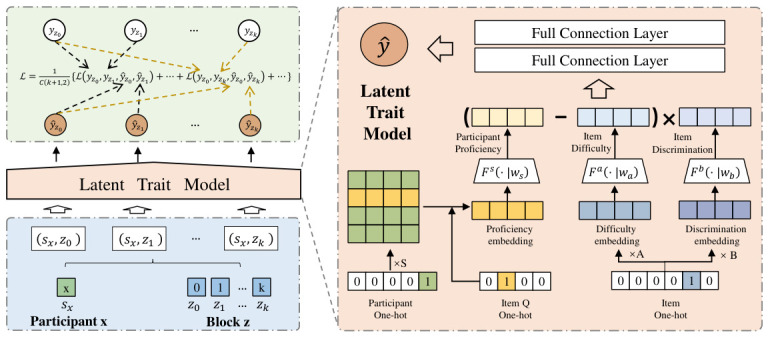
The framework of the proposed FCNLT model.

Figure 2 presents a schematic diagram of the FCNLT model. The model consists of five key components: embedded representation of participant and item factors, Non-linearity Mapping Layer, Interaction Function and predictive modeling, prediction of item scores within a block, and loss function and model training. This section elaborates on each of these five components in detail.

#### 3.2.1. Embedded Representation of Participant and Item Factors

In such latent trait modeling tasks, the input information typically consists of one-hot representation vectors of participants and items, and the purpose of constructing relevant features for participants and items is to establish an interaction function. The one-hot representation vectors for participants and items are denoted as $x_{s}\in {\left\{0,1\right\}}^{1\times N}$ and $x_{e}\in {\left\{0,1\right\}}^{1\times M}$, respectively. Considering that in forced-choice tests each item is associated with only one dimension, the dimension information of an item can also be represented using a one-hot representation vector, i.e., $x_{q}\in {\left\{0,1\right\}}^{1\times K}$.

The embedding representation of participant factors is defined as follows:

(1) $$s=x_{s}\times W_{s},$$

where $s\in R^{1\times K\times d}$ is a three-dimensional tensor representing the participant’s latent features across all *K* trait dimensions, with *d* being the embedding size, and $W_{s}\in R^{N\times K\times d}$ is the trainable matrix.

To ensure that the model respects the unidimensional nature of forced-choice items, the participant representation is restricted to the single trait dimension measured by the item. This is achieved as follows:

(2) $$h_{1}^{\mathrm{prof}}=s⊙\mathrm{unsqueeze}\left(x_{q}\right),$$

specifically, $\mathrm{unsqueeze}(x_{q})$ transforms the dimension vector from shape (1,K) to (1,K,1), enabling element-wise multiplication with s. Since $x_{q}$ is a one-hot vector, only the slice of s corresponding to the item’s measured dimension is retained, while all other slices become zero. The summation along the second dimension aggregates the *K* slices into a single vector, yielding $h_{1}^{\mathrm{prof}}\in R^{1\times d}$, the participant’s latent trait feature on the specific dimension relevant to the item.

As for each item, item difficulty and discrimination are defined as follows:

(3) $$h_{1}^{\mathrm{diff}}=x_{e}\times W^{\mathrm{diff}},$$

(4) $$h_{1}^{\mathrm{disc}}=x_{e}\times W^{\mathrm{disc}},$$

where $h_{1}^{\mathrm{diff}},h_{1}^{\mathrm{disc}}\in R^{1\times d}$ are the embedded representations of item difficulty and discrimination, respectively. $W^{\mathrm{diff}},W^{\mathrm{disc}}\in R^{M\times d}$ are the trainable matrices.

#### 3.2.2. Non-Linearity Mapping Layer

After obtaining the embedded representations, deep feature extraction is performed on the participant’s latent trait, item difficulty, and discrimination using a nonlinear activation function. This procedure introduces nonlinear representations of the features. Specifically, the transformations are defined as follows:

(5) $$h_{2}^{\mathrm{prof}}=\phi \left(W_{1}\times h_{1}^{\mathrm{prof}}+b_{1}\right),$$

(6) $$h_{2}^{\mathrm{diff}}=\phi \left(W_{2}\times h_{1}^{\mathrm{diff}}+b_{2}\right),$$

(7) $$h_{2}^{\mathrm{disc}}=\phi \left(W_{3}\times h_{1}^{\mathrm{disc}}+b_{3}\right),$$

where $W_{1}$, $W_{2}$, $W_{3}$ are the learnable matrices, $b_{1}$, $b_{2}$, $b_{3}$ are the corresponding bias terms, and ϕ denotes the activation function, which is the Sigmoid function in this paper.

#### 3.2.3. Interaction Function

The interaction function is defined as:

(8) $$x=h_{2}^{\mathrm{disc}}\times \left(h_{2}^{\mathrm{prof}}-h_{2}^{\mathrm{diff}}\right),$$

This is followed by a fully connected layer and an output layer:

(9) $$f_{1}=\phi \left(W_{4}\times x+b_{4}\right),$$

(10) $$y=\phi (W_{5}\times f_{1}+b_{5}).$$

To satisfy the monotonicity assumption, each element of $W_{4}$ and $W_{5}$ must be nonnegative. Here, $b_{4}$ and $b_{5}$ are the corresponding bias terms, ϕ is the activation function, and *y* represents the final output of the model.

#### 3.2.4. Prediction of Scores for Items in Item Block

In a forced-choice test, the relationships between scores are determined by participants’ preferences for items inside an item block. As a result, for participant $s_{n}$ and item block $z_{l}$, the model must be applied to each item in block $z_{l}$ to get a score prediction for each item within the block:

(11) $$y_{n,l}=\left\{y_{n,l_{1}},y_{n,l_{2}},\ldots ,y_{n,l_{t}}\right\}.$$

The ranking score of the items in the item block is given by:

(12) $$p_{n,l}=rank\left(y_{n,l}\right),$$

where $rank(y_{n,l})$ represents the ranked position of each probability value in the set $y_{n,l}$. The RANK item block type ranks based on probability values, with the smallest value assigned a rank of 1 and the largest a rank of *t*. In contrast, the ranking system for the MOLE item block type is slightly different: the smallest probability value is ranked first, the largest is ranked third, and the remaining values are ranked second.

#### 3.2.5. Model Optimization

This study assumes that participants’ choice processes among items follow Thurstone’s law of comparative judgment (Thurstone, 1927). This assumption is widely adopted in forced-choice personality assessment and underlies several established IRT models, including TIRT, MUPP-2PL, and BRB-IRT. According to this law, participants make independent pairwise comparisons among items within a block during ranking. In such pairwise comparisons, the higher-ranked item is viewed as a positive sample, and the lower-ranked item is treated as a negative sample. This means that the model must ensure that the predicted score of the higher-ranked item exceeds that of the lower-ranked item. This paper applies the BPR loss function (Rendle et al., 2009), extensively used in recommender systems, to forced-choice tasks. The loss function includes a weighting term depending on the score difference between the two items to better reflect their relative importance. The loss function for any pair of items $e_{i}$ and $e_{j}$ within a block is as follows:

(13) $$L\left(y_{i},y_{j},r_{i},r_{j}\right)=-\ln\left(\sigma \left(\frac{\lambda }{r_{i}-r_{j}}\cdot \left(y_{i}-y_{j}\right)\right)\right),$$

where $y_{i}$ and $y_{j}$ are the predicted scores for items $e_{i}$ and $e_{j}$, respectively, $r_{i}$ and $r_{j}$ are their ranked positions, λ is a weighting coefficient, and σ(·) is the Sigmoid activation function. This loss function aims to ensure that top-ranked items score higher than those ranked lower.

For the PICK item block type, the total loss is given by:

(14) $${\mathrm{loss}}_{\mathrm{PICK}}=\frac{1}{t-1}\sum_{j\neq p}L\left(y_{p},y_{j},r_{p},r_{j}\right),\;j=1,\ldots ,t,$$

where *p* denotes the index of the item selected by the participant as the most self-descriptive, and *t* is the number of items in the block. In the PICK format, only the t−1 pairs between the chosen item and each unchosen item are informative, so the loss is averaged over these pairs.

For the RANK item block type, the total loss is given by:

(15) lossRANK=1t2∑1≤i<j≤tL(yi,yj,ri,rj),

where t2 represents the number of item pairs in the block. This formula calculates the average loss across all item pairs.

For the MOLE item block type, the total loss is expressed as:

(16) lossMOLE=1t2−t−22∑1≤i<j≤tIMOLE(ri−rj)L(yi,yj,ri,rj),

where $I_{\mathrm{MOLE}}\left(\cdot \right)$ is the indicator function that equals 0 when $r_{i}=r_{j}$ and 1 otherwise. The ${\mathrm{loss}}_{\mathrm{MOLE}}$ is calculated by considering only pairs of items with different ranks, effectively excluding those with equal ranks.

## 4. Experiments and Results

In this study, we conduct comprehensive experiments to explore the following research questions:

- **RQ1:** How sensitive is FCNLT to its model parameters?
- **RQ2:** Does the FCNLT model outperform the baseline models?
- **RQ3:** How effective are the key components of the FCNLT model?
- **RQ4:** How interpretable are the latent trait and item parameters estimated by FCNLT?
- **RQ5:** What do the visualizations show about the interactions between participants, items, and dimensions?

### 4.1. Datasets

The experiments are carried out with three real-world datasets and one simulated dataset: BFI-P, MAP, BFI-R, and sim-mole.

- **BFI-P** is a forced-choice personality assessment based on the Japanese version of the Big-Five factor marker questionnaire (Bunji & Okada, 2020), which measures five personality traits: Emotional Stability, Extraversion, Agreeableness, Conscientiousness, and Imagination. Positively and negatively keyed items are treated as separate dimensions, resulting in a total of 10 dimensions and 25 item blocks. Each block contains two items from different dimensions (PICK format). The dataset includes responses from 499 participants.
- **MAP** is a business personality test developed by a company, consisting of 264 items that assess 24 dimensions across three aspects: Mental (M), Attitudes and Motivation (A), and People Skills (P). The test features 88 item blocks, with each block containing three items from different dimensions and a total of 11 items per dimension. Complete response data were collected from 1433 participants and organized in an item block format using RANK.
- **BFI-R** is a forced-choice personality assessment based on the Big Five Inventory 2 (Soto & John, 2017). It measures the five broad dimensions of Openness, Conscientiousness, Extraversion, Agreeableness, and Neuroticism using 60 items. In this study, positively and negatively scored items are treated as separate dimensions, resulting in a total of 10 dimensions and 20 item blocks, each containing three items from different dimensions. The dataset includes responses from 372 participants, also formatted as RANK item blocks.
- **sim-mole** dataset comprises 480 items across 24 dimensions and 120 item blocks, with each block containing four questions from different dimensions. Responses were generated for 1000 participants under specific conditions: the discrimination parameter was randomly sampled from a uniform distribution $U(0.75,1.5^{2})$, and the difficulty parameter from a normal distribution $N(0,0.5^{2})$. For each participant, a 24-dimensional vector of latent traits was generated from a multivariate normal distribution with a mean of 0 and a fixed covariance of 0.5 between dimensions. Participant responses were simulated using the MOLE response model, employing the Luce decision-making approach to calculate the probabilities of each response mode. Based on these probabilities, response matrices were generated using a multinomial distribution.

Table 3 provides detailed statistics for these four datasets.

### 4.2. Baselines

This study employs eight baseline methods, including MUPP-2PL, BRB-IRT, MF, RankNet, NCDM-R, KaNCD-R, CDND-R, and Random.

- **MUPP-2PL** (Morillo et al., 2016) is developed from the dominant response model and integrated with the MUPP framework (Stark et al., 2005) for effective selection of question-type data. Its formula is given by:
  (17) $$P\left(i>j|\theta_{q_{i}},\theta_{q_{j}}\right)=\phi \left(a_{i}\theta_{q_{i}}-a_{j}\theta_{q_{j}}+a_{i}b_{i}-a_{j}b_{j}\right),$$
  where ϕ denotes the sigmoid function, also known as the logistic function. Here, $a_{i}$ and $a_{j}$ are discrimination parameters for items, $\theta_{q_{i}}$ and $\theta_{q_{j}}$ represent the latent traits for items *i* and *j*, respectively, and $a_{i}b_{i}-a_{j}b_{j}$ serves as the intercept parameter derived from combining the *a* and *b* parameters in the 2PLM.
- **BRB-IRT** (Lee & Smith, 2020) is a Bayesian item block model (Bradlow et al., 1999) selected as the base model for forcing question selection. It incorporates the randomized question block effect parameter $\gamma_{l}$ into the item response function of MUPP-2PL, given the interdependence of topics within the block during parameter estimation. Its formula is:
  (18) $$P_{n,l}\left(i>j|\theta_{q_{i}},\theta_{q_{j}}\right)=\phi \left(a_{i}\theta_{q_{i}}-a_{j}\theta_{q_{j}}-\left(a_{i}b_{i}-a_{j}b_{j}\right)-\gamma_{n,l}\right),$$
  where $\gamma_{n,l}$ represents the random block effect for participant *n* on item block *l*, reflecting the influence of the dimensions measured by that block on the participant’s response.
- **MF** (F. Wang et al., 2020) is a matrix decomposition model used as a baseline in various cognitive diagnosis studies in education. It aligns participants and items with users and items in matrix decomposition, effectively predicting participant performance. However, it lacks explanatory power for cognitive diagnosis, as there is no clear mapping between elements in the trait vector and specific dimensions. Its formulation is given by:
  (19) $$y=MLP(h_{e}\circ h_{s}),$$
  where $h_{e}$ is the latent trait vector for the topic, $h_{s}$ is the latent trait vector for the subject, ∘ represents element-wise multiplication, and MLP consists of two fully connected layers and one output layer.
- **RankNet** (Burges et al., 2005) employs a neural network to predict relative rankings between pairs of items, aiming to minimize the difference between predicted and true rankings. It computes relative preferences by comparing pairs of items (positive and negative samples) and is trained using a cross-entropy loss function. The network structure of RankNet is similar to that of MF, as shown in Equation (19), but it uses the ReLU activation function in the fully connected layer, while the prediction layer remains Sigmoid. The probability of item *i* being ranked higher than item *j* is expressed as:
  (20) $$P\left(i>j\right)=\phi \left(y_{i}-y_{j}\right),$$
- **NCDM-R** (F. Wang et al., 2020) is a widely used deep learning-based cognitive diagnostic model that employs a multilayer perceptron to capture complex, higher-order interactions between subjects and topics. Its formulation is:
  (21) $$y=MLP(Q_{e}\circ \left(h_{s}-h^{diff}\right)\times h^{disc}),$$
  where $h^{diff}$ and $h^{disc}$ represent the difficulty of knowledge concepts and topic differentiation, respectively, and $h_{s}$ denotes the participant’s proficiency. The MLP consists of two fully connected layers and one output layer. The original NCDM only provides predicted scores for questions without ranking. To adapt it for forced-choice item types, the ranking probability formula from RankNet is applied, resulting in NCDM-R.
- **KaNCD-R** (F. Wang et al., 2022) builds on NCDM and incorporates the implicit relationships between knowledge concepts.
  (22) $$y=MLP(Q_{e}\circ \left(h_{s}\cdot l-h^{diff}\cdot l\right)\times h^{disc}),$$
  where l represents the vector of knowledge concepts associated with item *e*. For the modification of KaNCD, the same approach as in NCDM is adopted.
- **CDND-R** (Y. Zhang et al., 2025) uses an attention-like mechanism to capture the nonlinear dependencies between participants and items, while employing a discriminative interaction approach to exploit second-order interactions, thereby improving discriminative capability across different participant-item combinations. The CDND-R modifications take the same approach as those for NCDM.
- **Random** method predicts the participant’s probability of success on a topic using a uniform distribution Uniform(0,1). This generates a ranked score based on the magnitude of the predicted probabilities for each topic pair. In the interpretability analysis, the ability values of subjects across different dimensions are also randomly generated from the uniform distribution Uniform(0,1).

In summary, all models in this study receive the same PICK-2 pairwise comparison data as input. Regardless of whether the original block format is PICK, RANK, or MOLE, the response data are uniformly decomposed into PICK-2 item pairs before training. For FCNLT, the PICK-2 pairs originating from the same original block are organized together during loss computation solely to accommodate the ablation experiments in which a listwise loss function was tested. This grouping is used exclusively in the ablation variant (FCNLT_List), and the main FCNLT model computes the loss at the level of individual item pairs; hence, it does not alter the information content of the input or confer any additional informational advantage to the model. Therefore, all models are fairly comparable at the input level. Among the baseline models, MF was optimized using the BPR loss function, while the other models were optimized with the cross-entropy loss function.

### 4.3. Experimental Details

For parameter initialization, the Xavier method (Glorot & Bengio, 2010) was used, and the AdamW optimizer (Loshchilov & Hutter, 2019) was adopted for all models. The embedding dimension *d* was set to 64, with the nonlinear mapping layer sized at 256 and the fully connected layer at 128.

For a fair comparison, all baselines were tuned under a consistent protocol. The batch size was fixed at 128 across all models. The learning rate was selected from {0.0005,0.001,0.002,0.005,0.01} based on validation performance. Early stopping with a patience of 5 epochs was applied to MF and RankNet. For BRB-IRT, MUPP-2PL, and CDND-R, training was run for a fixed 100 epochs to ensure convergence. For NCDM-R and KaNCD-R, a fixed 150 epochs was used as preliminary experiments showed that these models required more iterations to converge under the gradient-based optimization framework. The final hyperparameters of FCNLT were determined through the sensitivity analyses reported in Section 4.5.

Experiments were conducted on a Linux server equipped with a 2.50 GHz Xeon Platinum 8255C CPU, a Tesla T4 GPU, and 32 GB of RAM. Each experiment was repeated 10 times, and the final results represent the average of these repetitions.

### 4.4. Evaluation Metrics

Before detailing the specific metrics, it is necessary to clarify the evaluation paradigm of this study. Traditional psychometric research often evaluates new models via parameter recovery on simulated data, assuming a predefined mathematical equation with absolute, observable parameters (Zheng et al., 2024). However, as the proposed FCNLT model is a data-driven deep neural network, participants’ latent traits and item features are modeled as dynamically optimized embeddings in a high-dimensional space. Therefore, following the established paradigm of neural cognitive diagnosis, the evaluation focus shifts from recovering absolute parameters to assessing the model’s predictive performance and generalization ability on unseen data (F. Wang et al., 2020, 2022).

The forced-choice personality test asks participants to rank all items in an item block based on their perceptions. To align with this mechanism, this study evaluates the model’s predictive performance using two ranking-based metrics: pairwise rank accuracy (PRA) and listwise rank accuracy (LRA).

**PRA** measures the consistency between the model’s predicted rankings and the actual rankings across all possible pairs of items. Its specific expression is given as follows for different item block types:

(23) $${\mathrm{PRA}}_{\mathrm{PICK}}=\frac{1}{L}\sum_{l=1}^{L}\frac{1}{t-1}\sum_{j\neq p}I_{\mathrm{PRA}}\left(\left(y_{p}-y_{j}\right)\left(r_{p}-r_{j}\right)>0\right),\;j=1,\ldots ,t$$

(24) PRARANK=1L∑l=1L1t2∑1≤i<j≤tIPRA(yi−yj)(ri−rj)>0,

(25) PRAMOLE=1L∑l=1L1t2−t−22∑1≤i<j≤tIMOLE(ri−rj)IPRA(yi−yj)(ri−rj)>0,

where *L* is the total number of item blocks, and $I_{\mathrm{PRA}}\left(\cdot \right)$ is an indicator function that equals 1 when the predicted ordering aligns with the true ordering and 0 otherwise.

**LRA** evaluates the accuracy of the model’s predicted ranking of the entire item block, using the following formula:

(26) $$\mathrm{LRA}=\frac{1}{L}\sum_{l=1}^{L}I_{\mathrm{LRA}}\left(p_{l}=r_{l}\right),$$

where $I_{\mathrm{LRA}}\left(\cdot \right)$ is the indicator function, which takes the value of 1 if and only if the predicted block ordering $p_{l}$ is perfectly identical to the actual block ranking $r_{l}$, and 0 otherwise.

### 4.5. Hyperparameter Analysis (RQ1)

The hyperparameters in FCNLT include λ, batch size, and training set ratio. This section examines how these hyperparameters affect FCNLT’s performance and assesses its robustness.

#### 4.5.1. The Effect of λ

**Figure 3:**
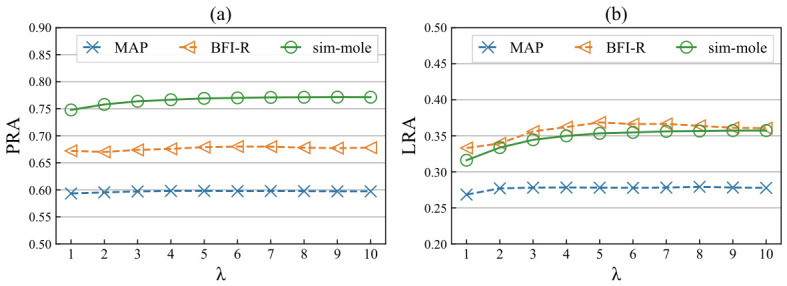
The impact of λ. (**a**) PRA; (**b**) LRA.

The analysis of the hyperparameter λ is presented in Figure 3. For the MAP dataset, both the PRA and LRA show minimal variation with changes in λ, indicating low sensitivity, with optimal performance achieved at λ=8. In the BFI-R dataset, PRA and LRA initially increase before leveling off, peaking around λ=5. This suggests that a moderate λ value enhances model optimization. Conversely, in the sim-mole dataset, PRA and LRA consistently increase with λ, particularly between λ=1 and λ=5, where performance improvements are most pronounced. However, for λ>5, the growth in PRA and LRA diminishes and stabilizes, indicating that larger λ values yield limited performance gains.

Overall, the response to λ varies across datasets: a moderate λ optimizes performance in real-world datasets, while a larger λ significantly improves the simulated dataset’s performance, albeit with diminishing returns. This highlights the need for tailored λ values to optimize model performance across different datasets.

#### 4.5.2. The Effect of Batch Size

**Figure 4:**
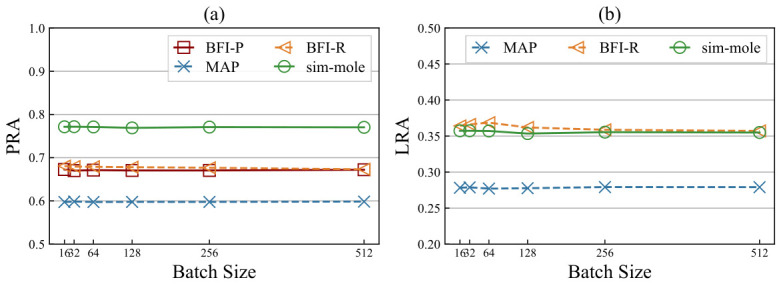
The impact of batch size. (**a**) PRA; (**b**) LRA.

The analysis of batch size is illustrated in Figure 4, where the result for each batch size corresponds to the best-performing learning rate among those tested. Because BFI-P employs the PICK-2 format, its PRA and LRA are mathematically equivalent; hence only the PRA curve is presented (and likewise in the subsequent analyses). For the MAP and BFI-P datasets, both PRA and LRA remain stable across all batch sizes, indicating that model performance is insensitive to batch size on these datasets. For the BFI-R and sim-mole datasets, performance also shows no clear trend with changing batch size, with the metrics fluctuating within a narrow range. Overall, the sensitivity to batch size is low across all four datasets, demonstrating that FCNLT is robust to variations in batch size and can maintain consistent performance over a wide range of batch sizes.

#### 4.5.3. The Effect of Train Set Ratio

**Figure 5:**
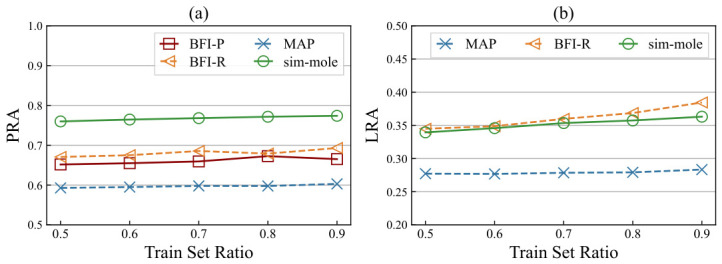
The impact of train set ratio. (**a**) PRA; (**b**) LRA.

The analysis of the training set ratio is presented in Figure 5. The MAP dataset demonstrates strong stability, remaining largely unaffected by variations in the training set ratio. BFI-P reaches its optimal performance at a training ratio of 0.8, with a slight decline at 0.9, though the overall variation is limited. In contrast, both the BFI-R and sim-mole datasets show a gradual performance increase with higher training set ratios. Overall, all four datasets achieve satisfactory performance even at the lowest training ratio of 0.5, demonstrating that FCNLT can learn effectively from relatively limited training data.

The final hyperparameters for the BFI-P, MAP, BFI-R, and sim-mole datasets are as follows: λ values of 1, 8, 5, and 10; batch sizes of 16, 256, 64, and 32; and learning rates of 0.001, 0.01, 0.005, and 0.0005 respectively. For each dataset, participant response data were first randomly divided into a training set and a test set at an 8:2 ratio by item block. The training set was then further split into an 8:2 ratio to create a validation set for model selection and hyperparameter tuning. An early-stopping mechanism was implemented on the validation set: training stops and the best model is saved if evaluation metrics do not improve for 5 consecutive iterations. All reported results were evaluated on the independent test set.

### 4.6. Performance Prediction Task for Participants (RQ2)

To systematically evaluate the proposed model’s performance, the subsequent analyses focus on three key dimensions: predictive accuracy, computational efficiency, and convergence dynamics during training.

#### 4.6.1. Predictive Performance Analysis

The results of the participant performance prediction task are summarized in Table 4. The table reports the mean ± standard deviation over 10 independent runs, with statistical significance determined by paired Wilcoxon signed-rank tests and Holm–Bonferroni correction for multiple comparisons (see the table note for details). The analysis leads to the following conclusions:

(1) **FCNLT demonstrates significant superiority in LRA.** Across the MAP, BFI-R, and sim-mole datasets, the LRA of FCNLT was significantly higher than those of all baseline models. In terms of PRA, FCNLT achieved the highest numerical values on the BFI-P, MAP, and sim-mole datasets. While it significantly outperformed all baselines on sim-mole, its advantage over the second-best model (NCDM-R) on BFI-P was not statistically significant. Similarly, on MAP, the marginal PRA differences between FCNLT and both NCDM-R and KaNCD-R were likewise non-significant. On BFI-R, FCNLT’s PRA was slightly lower than those of NCDM-R and KaNCD-R, but the three models did not differ significantly, indicating comparable performance levels.

(2) **Among the baseline models, NCDM-R exhibited the strongest overall performance.** It attained the highest PRA on BFI-R and ranked second in PRA on BFI-P and MAP, as well as in both PRA and LRA on sim-mole; in all of these cases, the differences from FCNLT were not statistically significant. KaNCD-R tied with NCDM-R for the best PRA on BFI-R and also showed no significant difference from FCNLT. Thus, NCDM-R and KaNCD-R represent the most competitive benchmarks for FCNLT, particularly with respect to PRA.

(3) **Model performance is moderated by block format and data characteristics.** On the MAP and BFI-R datasets, which use a RANK-3 format, neural network-based models generally outperformed traditional statistical models such as MUPP-2PL and BRB-IRT. On the sim-mole dataset (MOLE-4 format), traditional statistical models exhibited strong PRA performance yet remained significantly lower than FCNLT. On the BFI-P dataset (PICK-2 format), neural network models also demonstrated favorable predictive ability. This pattern suggests that different methods hold distinct advantages under different block formats.

#### 4.6.2. Computational Efficiency Analysis

Beyond predictive accuracy, computational efficiency is also a critical metric of model practicality, particularly in high-dimensional forced-choice assessment contexts. The computational efficiency of all models is summarized in Table 5, which reports the number of parameters, GPU memory consumption, per-epoch training time, the optimal epoch, and the time required to reach this peak state (Time to Best). Time to Best is calculated as the mean per-epoch time multiplied by the optimal epoch, thereby eliminating the influence of varying stopping criteria. All time-related metrics are presented as the mean ± standard deviation across 10 independent runs. The analysis yields the following findings:

(1) **Traditional statistical models feature small parameter footprints, negligible GPU memory consumption, and short per-epoch time, but their slow convergence hampers overall training efficiency.** The GPU memory usage of MUPP-2PL and BRB-IRT is less than 4 MB, substantially lower than that of the neural network models. However, they require a large number of epochs to reach optimal performance across all datasets, with even more epochs needed on the higher-dimensional MAP and sim-mole datasets, leading to markedly longer Time to Best compared with the neural models. This indicates that, in high-dimensional settings, the computational burden of traditional models arises not only from the estimation algorithm itself but also from their inherently slow convergence.

(2) **FCNLT trades a larger parameter count for extremely fast convergence, achieving a favorable balance between efficiency and predictive accuracy.** FCNLT converges within only 1–8 epochs on all datasets, substantially faster than baseline models such as NCDM-R and KaNCD-R. In terms of Time to Best, FCNLT is on the same order of magnitude as the most efficient but less accurate models MF and RankNet on high-dimensional datasets, and it is far superior to the traditional models. On BFI-P and BFI-R, its training time is slightly longer than that of MF and RankNet; however, given its significantly superior predictive capabilities, this additional overhead is entirely acceptable. Furthermore, although the GPU memory consumption of FCNLT is slightly higher than that of other neural models, it remains well within the ample capacity of modern GPUs.

(3) **The neural network models exhibit pronounced divergence in training efficiency.** MF and RankNet converge rapidly with short training times, but their predictive accuracy is substantially lower than that of FCNLT. NCDM-R and KaNCD-R, despite their competitive predictive performance, require considerably more epochs to converge than FCNLT across all datasets, resulting in correspondingly longer Time to Best. CDND-R falls between the two extremes in both efficiency and performance. In contrast, FCNLT maintains state-of-the-art predictive performance while achieving consistently faster convergence and acceptable training overhead.

#### 4.6.3. Convergence Dynamics Analysis

**Figure 6:**
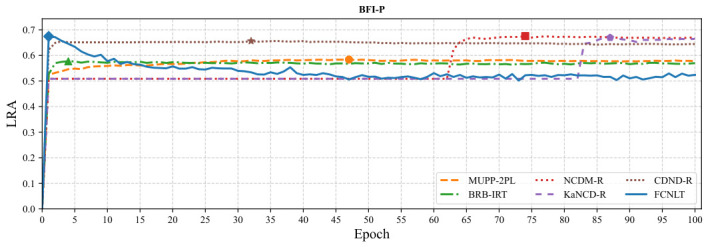
Trend of LRA over Training Epochs (BFI-P Datasets).

**Figure 7:**
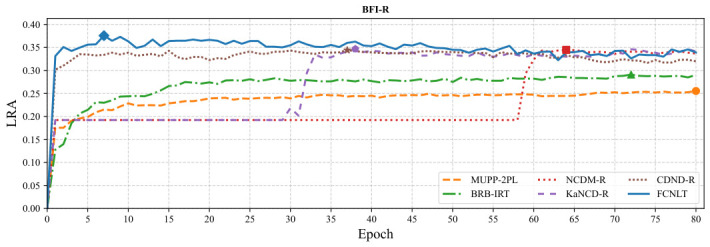
Trend of LRA over Training Epochs (BFI-R Datasets).

**Figure 8:**
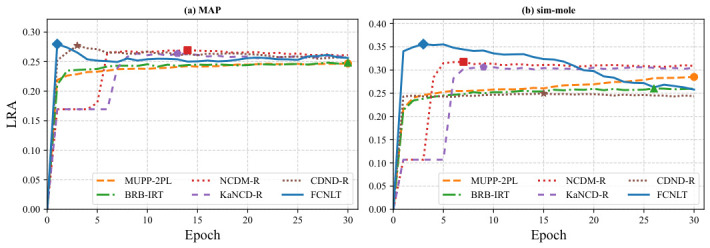
Trend of LRA over Training Epochs (MAP and sim-mole Datasets).

To further examine the convergence dynamics of different models during training, we tracked the LRA metric of MUPP-2PL, BRB-IRT, NCDM-R, KaNCD-R, CDND-R, and FCNLT on the test set across training epochs. Since BFI-P employs the PICK-2 format, its PRA is mathematically equivalent to LRA; for brevity, we refer to it as LRA in the corresponding figures. The selected epoch ranges are as follows: the first 100 epochs for BFI-P, the first 80 epochs for BFI-R, and the first 30 epochs for MAP and sim-mole. The results are presented in Figure 6, Figure 7 and Figure 8. The main observations are as follows: (1) **FCNLT and CDND-R exhibit consistently fast convergence across all datasets**, reaching or closely approaching the optimal LRA at an early training stage. However, the final LRA of CDND-R is markedly lower than that of FCNLT, indicating that although the two models converge at a similar speed, FCNLT achieves a substantially higher predictive ceiling. (2) **NCDM-R and KaNCD-R display a pronounced convergence stagnation phase during early training.** The optimization of these two models virtually stagnates in the initial epochs, with the test LRA appearing as a flat line in the convergence plots before it begins to improve. While this early-stage stagnation is relatively weaker on the MAP and sim-mole datasets, it remains particularly prominent on the two smaller BFI datasets. (3) **MUPP-2PL and BRB-IRT exhibit the slowest convergence.** Within the observed epoch ranges, their LRA increases steadily but never surpasses that of the neural network models, and their final LRA values remain the lowest overall.

Integrating the analyses of predictive accuracy, computational efficiency, and convergence dynamics, FCNLT demonstrates a consistent and significant advantage in LRA. It performs comparably to the strongest baselines in PRA while achieving optimal predictive accuracy with minimal training epochs and low time overhead. Furthermore, these findings indicate that neural network models developed for conventional educational contexts do not readily transfer to forced-choice personality assessments. This underscores the necessity of designing models specifically tailored to the unique response mechanisms of non-cognitive forced-choice tests.

### 4.7. Ablation Experiments (RQ3)

To assess the contribution of each model component to overall performance, ablation experiments were performed, with four variants created:

- **FCNLT_EB**: This variant removes the non-linearity mapping layer, directly constructing the interaction function using $h_{2}^{\mathrm{prof}}$, $h_{2}^{\mathrm{diff}}$, and $h_{2}^{\mathrm{disc}}$ instead of $h_{1}^{\mathrm{prof}}$, $h_{1}^{\mathrm{diff}}$, and $h_{1}^{\mathrm{disc}}$ to assess the impact of the non-linearity mapping layer.
- **FCNLT_BPR**: This variant uses the original BPR loss function but excludes the difference in ranked scores as a weighting term. The formula for calculating the original BPR loss is:
  (27) $$L\left(y_{i},y_{j},r_{i},r_{j}\right)=-\ln\sigma \left(\mathrm{sgn}\left(r_{i}-r_{j}\right)\cdot \left(y_{i}-y_{j}\right)\right)$$
  where sgn is the **sign** function, which returns the sign of the input.
- **FCNLT_List**: This variant replaces BPR loss with listwise loss, which calculates the logarithmic difference between the predicted scores of each item in the block and their subsequent output scores. The predicted scores are logarithmized and compared with the exponential values of the remaining items, with the negative average taken. The specific formula for listwise loss is:
  (28) $$L=-\frac{1}{T}\sum_{i=1}^{T}\left(y_{n,i}-\log\left(\sum_{j=i}^{T}e^{y_{n,j}}\right)\right),$$
  where *T* is the number of items in the block and $y_{n,i}$ is the predicted score for the *i*-th item. This loss function promotes global consistency in sequence predictions while enhancing the model’s focus on overall ranking.
- **FCNLT_MO**: To investigate the impact of the monotonicity assumption on model performance, FCNLT_MO removes the non-negative constraints on $W_{4}$ and $W_{5}$ in Equations (9) and (10).

Table 6 presents the ablation experiment results. The key findings include:

(1) **The weighted BPR loss plays an irreplaceable role in the model’s predictive performance.** FCNLT_BPR and FCNLT_List both perform significantly worse than the full FCNLT across all metrics on the MAP, BFI-R, and sim-mole datasets. The former result indicates that the score-difference weighting term effectively enhances the model’s sensitivity to preference strength, while the latter demonstrates that the BPR loss framework is better suited than listwise loss for modeling relative preferences in forced-choice data. The performance degradation of FCNLT_List is particularly pronounced on the MOLE-format sim-mole dataset, further highlighting the limitations of listwise loss in handling incomplete ranking tasks. On the BFI-P dataset, both ablation variants perform slightly worse than the full FCNLT, but the differences do not reach statistical significance. This is likely because the PICK format only requires selecting the most self-descriptive item, leaving no preference distinctions among the unselected items; this results in fewer informative pairwise comparisons, consequently reducing the performance gap among different loss functions.

(2) **Removing the monotonicity constraint has no significant impact on predictive performance.** Across all datasets and metrics, the differences between FCNLT_MO and the full FCNLT are not statistically significant. In fact, FCNLT_MO achieves values comparable to or even slightly better than those of FCNLT on several metrics. Therefore, FCNLT_MO is introduced as a comparison model in the subsequent interpretability analyses.

(3) **The contribution of the non-linear mapping layer varies across datasets and metrics.** FCNLT_EB achieves the best PRA on the BFI-P dataset, outperforming the full FCNLT, although the difference is not statistically significant. On MAP and BFI-R, its LRA is significantly lower than that of the full model, whereas on the PRA metric and on the sim-mole dataset, no significant differences are observed. These findings suggest that the impact of the non-linear mapping layer depends on the specific data format and evaluation metric, but its retention helps safeguard the model’s overall performance across different scenarios.

In summary, although the contributions of individual components vary across data formats, the weighted BPR loss demonstrates clear advantages over both the original BPR and listwise loss in most settings; the monotonicity constraint does not affect predictive performance; and the influence of the non-linear mapping layer depends on the evaluation context. Given FCNLT’s general-purpose design goal of accommodating multiple forced-choice formats, all of the above components should be retained.

### 4.8. Parameter Interpretability Analysis (RQ4)

To evaluate whether the parameters learned by FCNLT carry meaningful psychometric interpretations, two complementary analyses are conducted: parameter recovery on simulated data and degree of agreement (DOA) analysis on real-world datasets.

#### 4.8.1. Parameter Recovery on Simulated Data

The sim-mole dataset provides known true generating parameters for both latent traits and items, offering a benchmark for assessing the quality of FCNLT’s parameter estimates.

For both participant trait parameters and item parameters, FCNLT generates the corresponding latent representations through the non-linear mapping layer (Equations (5)–(7)). Specifically, the participant trait representation $h_{2}^{\mathrm{prof}}$ is obtained by applying the non-linear mapping to the participant embedding. The item discrimination $h_{2}^{\mathrm{disc}}$ and difficulty $h_{2}^{\mathrm{diff}}$ are obtained analogously from their respective embeddings. For participant traits, the vector $h_{2}^{\mathrm{prof}}$ is further averaged across the multi-head representation dimension to produce a scalar trait score for each dimension. For item parameters, to evaluate whether these high-dimensional vectors encode the true scalar information, we employ cross-validated ridge regression to linearly map the high-dimensional representations to scalars, and then compute the Pearson correlation with the true values. The aggregated results over 10 independent runs are summarized in Table 7.

As shown in Table 7, all three types of parameters are robustly recovered. The recovered parameters exhibit statistically significant correlations (p<0.001) with their true generating values across all dimensions. Notably, the model demonstrates the highest fidelity in recovering item discrimination, followed by latent traits and item locations. These results indicate that the latent embeddings learned by FCNLT capture meaningful individual differences and item characteristics. The model does not merely optimize for ranking accuracy, but its internal parameters indeed encode interpretable psychometric information.

#### 4.8.2. Degree of Agreement Analysis

Monotonicity is a fundamental prerequisite for latent trait models, and the interpretability of a model is dependent on its adherence to this assumption. According to the monotonicity assumption (Rosenbaum, 1984), a participant’s potential trait level in a given dimension should be positively correlated with their scores on related items. However, forced-choice tests only provide ranked scores between items, so specific response scores are unavailable. As a result, this study defines the interpretability of the forced-choice test model as follows: a participant with a higher latent trait level in a specific dimension is more likely to assign a higher rank to items measuring that dimension within a block.

Consistency is used as an evaluation metric for sorting performance, and the Degree of Agreement (DOA) is defined as follows:

(29) DOA=1K2∑1≤a<b≤KI(Faprof−Fbprof)(Sa−Sb)>0,

where $F_{a}^{\mathrm{prof}}$ and $F_{b}^{\mathrm{prof}}$ represent the latent trait level values of the participant in dimensions $c_{a}$ and $c_{b}$, respectively, and $S_{a}$ and $S_{b}$ denote the sums of ranked scores for the items in those dimensions. $I_{\mathrm{DOA}}\left(\cdot \right)$ is an indicator function that takes the value of 1 if the potential trait level on dimension $c_{a}$ is greater than that on dimension $c_{b}$ and the corresponding sum of ranking scores for dimension $c_{a}$ is also greater than that for dimension $c_{b}$; otherwise, it takes the value of 0.

**Figure 9:**
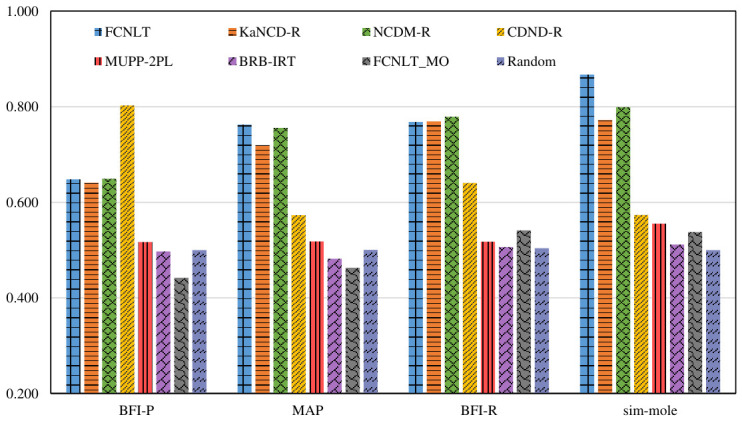
DOA results of models on four datasets.

The FCNLT model is evaluated against several benchmark models, including KaNCD-R, NCDM-R, CDND-R, MUPP-2PL, BRB-IRT, FCNLT_MO, and Random. Notably, MF and RankNet are excluded from the comparison due to the lack of clear correspondences between their latent characteristics and knowledge concepts. Figure 9 illustrates the experimental results: (1) FCNLT, KaNCD-R, and NCDM-R consistently rank among the top models in DOA across all four datasets. While CDND-R achieves the highest DOA on the BFI-P dataset, FCNLT performs the best on the MAP and sim-mole datasets. On the BFI-R dataset, NCDM-R slightly outperforms FCNLT and KaNCD-R. Across all scenarios, these top-performing neural network models remain substantially above the random baseline. (2) Although also a deep learning model, CDND-R yields lower DOA values than other deep learning models on the MAP, BFI-R, and sim-mole datasets, while still exceeding the random baseline. (3) The DOA values of MUPP-2PL and BRB-IRT remain close to the random baseline, indicating limited consistency in ranking latent traits. (4) The variant without the monotonicity assumption, FCNLT_MO, produces DOA values near the random baseline across all datasets, a finding consistent with prior studies (F. Wang et al., 2020, 2022). This confirms that the monotonicity constraint is essential for maintaining the logical consistency of the learned latent space, even though it does not affect predictive performance.

In summary, the parameter recovery analysis demonstrates that FCNLT’s latent embeddings capture meaningful individual differences and item characteristics. Concurrently, the DOA analysis confirms that the monotonicity constraint is critical for preserving the interpretability of these parameters. Importantly, because item parameters are globally shared and all participant embeddings are learned within the same optimization framework, the trait scores estimated by FCNLT are not constrained to sum to a constant across dimensions. As a result, they carry normative properties that support inter-individual comparisons, similar to those produced by traditional forced-choice IRT models. This property has been empirically validated on simulated data with known true parameters; its generalization to real-world datasets awaits further investigation using external criteria, as discussed in Section 5.3. Together, these findings provide converging evidence that FCNLT learns psychometrically meaningful representations, rather than merely optimizing for ranking accuracy.

### 4.9. Case Study (RQ5)

**Figure 10:**
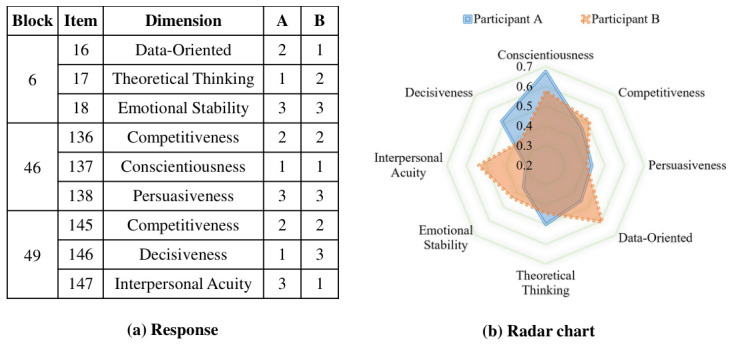
Diagnosed results of two participants on MAP.

Figure 10 illustrates a specific case of two participants assessed by the FCNLT model in the MAP dataset. The left side of the figure presents the participants’ responses across three item blocks, including the corresponding item dimensions and ranked responses, where “1” indicates the most self-descriptive, “2” the second most, and “3” the least. The right side displays radar charts showing the participants’ estimated latent trait levels in these dimensions.

Two key observations emerge from the visual analysis: (1) Participants with higher latent trait values in a dimension are more likely to rank items measuring that dimension favorably. For example, in item block 46 (which included items 136, 137, and 138, which correspond to the dimensions of Competitiveness, Conscientiousness, and Persuasiveness, respectively), both participants ranked the items as “2, 1, 3”. This indicates that they perceived item 137 (Conscientiousness) as the most self-descriptive. (2) Although Participants A and B exhibited identical rankings in item block 46, there are substantial differences in their estimated latent trait values for the pertinent dimensions. Specifically, Participant A obtained higher trait estimates than Participant B on the dimensions of Conscientiousness and Persuasiveness, but a lower estimate on Competitiveness. This case demonstrates the FCNLT model’s ability to estimate latent trait levels at the individual level as well as its effectiveness in inferring individual differences in ability traits using response ranking.

## 5. Discussion

This study aims to address the computational efficiency challenges faced by traditional latent trait models when handling high-dimensional forced-choice data and to overcome the limited adaptability of existing deep learning models from the educational domain to forced-choice scenarios. To this end, we propose a Neural Latent Trait Model specifically designed for forced-choice tests. Empirical results across four datasets comprehensively demonstrate the model’s effectiveness: In terms of LRA, FCNLT significantly outperforms all baseline models on all four datasets; for PRA, FCNLT exhibits a substantial advantage on the simulated dataset (sim-mole) while performing on par with the strongest baseline models on the real-world datasets. Regarding computational efficiency, despite its larger parameter space, FCNLT converges extremely fast, substantially outperforming traditional statistical models. Furthermore, parameter recovery and DOA analyses mutually confirm that the latent representations learned by FCNLT hold robust psychometric meaningfulness. In conclusion, FCNLT successfully achieves an effective balance among predictive accuracy, computational efficiency, and interpretability.

These findings suggest that the design of latent trait models must be tailored to specific test formats and data characteristics (Li et al., 2025). Although traditional statistical models theoretically align with the forced-choice mechanism, they suffer from a severe “curse of dimensionality” (Nie et al., 2024; Zheng et al., 2024). On the other hand, deep learning models originating from the educational domain rely on absolute scoring mechanisms (F. Wang et al., 2020, 2022). Directly migrating such models to forced-choice scenarios, which inherently generate “ipsative data,” inevitably leads to a misalignment between the model architecture and the true data-generating mechanism. To bridge this gap, FCNLT not only leverages deep neural networks for high-dimensional feature extraction but also innovatively introduces a weighted BPR mechanism into the loss function. This design maximizes the discriminability of participants’ relative preferences for different items, enabling the model architecture to closely match the inherent relative scoring properties of forced-choice tests. Consequently, this study validates the applicability of ranking-based deep learning algorithms in non-cognitive assessments.

### 5.1. Model Performance and Interpretability Analysis

Regarding predictive performance, FCNLT exhibited differential performance patterns on PRA and LRA. On the real-world datasets, FCNLT’s PRA was competitive with the strongest baselines, whereas its LRA significantly outperformed all baselines. This discrepancy may stem from the different sensitivities of the two metrics: PRA measures local pairwise rankings, whereas LRA requires the overall ranking within an item block to be entirely correct. The score difference weight term, introduced via the weighted BPR loss, enhanced the FCNLT’s global grasp of relative preference intensities among items within a block, thereby yielding a more consistent advantage in LRA. Under the PICK-2 format (BFI-P), which contains only two items per block, PRA is equivalent to LRA, and the pairwise ranking capabilities of the models were relatively close.

Ablation studies further revealed the adaptability between loss functions and item formats. Under the RANK format, the weighted BPR improved LRA compared to the original BPR, indicating that the original BPR only distinguishes between positive and negative samples without fully utilizing preference intensity information (Rendle et al., 2009); the weight term, by introducing score differences between ranked items, enhances the model’s fitting of the relative importance of options. Conversely, under the MOLE format, because the task involves numerous partial rankings and tied rankings, listwise losses were severely disrupted, whereas pairwise comparison methods demonstrated greater robustness. Under the PICK format, the discriminability among different loss functions was marginal, primarily because this format provides less valid pairing information, which limited the effectiveness of the weighting mechanism.

Regarding computational efficiency and convergence speed, the performance of different models was directly influenced by their network architectures. MF and RankNet have simple structures and extremely short per-epoch training times, but their predictive performance was sub-optimal. Networks inherited from cognitive diagnosis models, such as NCDM-R, involve complex interaction computations and lack architectural optimization for forced-choice data; consequently, they experienced convergence stagnation lasting dozens of epochs during early training, resulting in longer overall training times. In contrast, although FCNLT introduces additional trainable parameters, its structural design highly aligns with the characteristics of forced-choice tests. It rapidly converged to the optimal LRA within 1 to 8 epochs, and the time required to reach peak performance was on the same order of magnitude as the most lightweight models.

Regarding the baseline adaptation strategy, paired Wilcoxon signed-rank tests with Holm–Bonferroni correction for multiple comparisons indicated that replacing the loss function of the best-performing NCDM-R with the BPR loss (NCDM-bpr) did not yield significant performance differences (see Appendix A.1 for detailed results). Combined with the suboptimal performance of listwise losses in the ablation study, this suggests that uniformly adopting the RankNet pairwise probability formula as the baseline adaptation strategy is reasonable and fair, ensuring that performance differences can be genuinely attributed to the model architectures themselves.

In terms of interpretability, parameter recovery analysis demonstrated that the correlations between the parameters estimated by FCNLT and their true values all reached statistical significance. This stable correspondence enabled FCNLT to successfully overcome the inherent “ipsativity” limitation of forced-choice data. Its estimated trait scores are not forcibly constrained to a constant sum across dimensions, thereby recovering absolute latent trait levels with normative meaning and directly supporting inter-individual comparisons.

Furthermore, the results of the DOA metric, redefined based on the principle that a higher latent trait level corresponds to a greater probability of receiving a higher ranking, showed that FCNLT achieved excellent ranking consistency across most datasets. Importantly, the variant FCNLT_MO, which removed the monotonicity assumption, approached the random baseline on DOA without exhibiting a significant drop in predictive performance. This finding clarifies the core value of the monotonicity constraint: its contribution to predictive accuracy is negligible, but it is indispensable for maintaining the positive correlation between latent traits and item rankings, thus ensuring the psychometric interpretability of the model parameters (F. Wang et al., 2020).

### 5.2. Theoretical and Practical Implications

First, this study integrates the non-linear representational capacity of deep neural networks with the optimization objective of learning-to-rank. By introducing a relative comparison mechanism at the loss function level to handle ipsative forced-choice data, FCNLT breaks through the framework limitations of traditional IRT models, providing a novel computational paradigm for the estimation of latent traits. This indicates that ranking-based deep learning algorithms, originally developed for recommendation systems and information retrieval, can be effectively adapted to the psychometric domain, specifically when the data-generating process involves relative preferences rather than absolute scores.

Second, the findings emphasize the importance of matching model design with test characteristics. Models developed for absolute, independent item responses in educational assessments are difficult to migrate directly to forced-choice scenarios; this is evidenced by the slow convergence and sub-optimal performance of several neural baseline models. This suggests that future model development for forced-choice assessments should explicitly account for the ipsative nature of the data, the unidimensionality of items, and the inherent pairwise comparison process within forced-choice blocks, rather than simply relying on general architectures designed for traditional educational cognitive diagnosis models.

Third, at the applied level, the fast convergence and computational efficiency of FCNLT make it suitable for various practical assessment scenarios. In personnel selection, where large numbers of applicants need to be evaluated across multiple trait dimensions under tight time constraints, FCNLT can be trained on the current applicant sample and provide trait estimates within minutes, whereas traditional IRT models based on MCMC or EM algorithm variants may require dozens of hours to converge. In career counseling, FCNLT can generate normative latent trait scores that support inter-individual comparisons, enabling practitioners to provide quantitative feedback on clients’ relative strengths and weaknesses across multiple vocational interest dimensions. In large-scale psychological surveys, when both the number of participants and the number of trait dimensions are large, FCNLT offers a scalable alternative to traditional estimation methods, which face prohibitive computational costs in high-dimensional contexts.

### 5.3. Limitations

This study has several limitations that warrant attention in future research.

First, due to the relatively small sample sizes and number of item blocks in the BFI-R and BFI-P datasets, the data scale may impose certain constraints on the robustness of the deep learning model’s performance evaluation. Although our supplementary experiments using 5-fold cross-validation confirmed that the model’s performance was not overestimated under the current data split (see the Appendix A.2), the statistical significance across different splits exhibited some fluctuation under extreme conditions of severe data sparsity (e.g., the PICK-2 format in BFI-P). Future research should further validate the generalization ability of the proposed model on larger-scale, high-dimensional forced-choice personality datasets.

Second, FCNLT currently adopts a one-hot embedding paradigm for participant representation, which implies that the model essentially operates under a transductive learning framework. Once the model is trained, it cannot directly perform immediate scoring for new participants via forward propagation; instead, it requires re-optimizing the participant parameters. This limitation stems from the prevalent use of the one-hot encoding paradigm within the deep learning community. Future work could explore extending the model to an inductive inference architecture (W. Gao et al., 2024) by introducing inference networks or user-side covariates, thereby resolving the “cold start” problem for new users.

Third, constrained by the lack of external criteria in the empirical data, this study did not conduct an in-depth validation of the external validity of the model parameters. Future research could concurrently collect data using corresponding Likert scales or other behavioral criteria to examine the convergent validity of the scores estimated by FCNLT. This would provide more direct empirical support for the accuracy and psychometric meaningfulness of the model in recovering normative scores.

Finally, the performance of the proposed model was primarily validated on non-clinical personality traits; its applicability in clinical assessment contexts, which demand exceptionally high diagnostic precision and established norms, requires further investigation.

### 5.4. Future Research

First, future research could incorporate additional influencing factors, such as response time (RT) and item semantic information. Response time not only reflects participants’ cognitive processes but is also closely related to their cognitive abilities and the difficulty of item ranking; meanwhile, the semantic information within the item text directly impacts item characteristics. Incorporating these factors into the model would help enhance the precision and interpretability of the assessment.

Second, exploring modeling approaches based on Graph Neural Networks (GNNs) presents a promising avenue. Existing studies indicate that in cognitive diagnosis within the educational domain, the relationships among participants, items, and dimensions can be structured as graphs (W. Gao et al., 2021). Similarly, in forced-choice tests, GNNs can be utilized to characterize the intra-layer and inter-layer interactions between participants and items, thereby mining deeper latent features.

Finally, the application scenarios of forced-choice tests can be further expanded. By integrating deep learning algorithms to design intelligent item selection strategies, the personalization and adaptability of the assessments can be enhanced, ultimately realizing intelligent computerized adaptive forced-choice assessments for non-cognitive traits (e.g., personality and motivation).

## 6. Conclusions

This study proposed the FCNLT model tailored to the data characteristics of forced-choice tests. By incorporating dimension embedding compression, nonlinear feature mapping, and a weighted BPR loss function, the model effectively addresses the computational efficiency issues of traditional psychometric models on high-dimensional forced-choice data. Furthermore, it overcomes the limited adaptability of existing deep learning models when directly transferred to forced-choice scenarios. Experimental results on four datasets demonstrate that FCNLT outperforms traditional statistical models and deep learning baselines in overall prediction accuracy, convergence speed, and interpretability. These findings validate the feasibility of combining deep neural networks with ranking loss optimization for handling ipsative data, offering a new technical pathway for large-scale, high-dimensional personality assessment and the measurement of non-cognitive traits.

## Figures and Tables

**Table 1:** Comparison of the three forced-choice formats.

Item Block	PICK	RANK	MOLE
A. I stay calm under pressure		3	×
B. I enjoy helping others	✓	1	✓
C. My happiness is contagious		2	

Note: “✓” and “1” indicate that the participant considers the statement most self-descriptive; “2” indicates the second most self-descriptive; “×” and “3” indicate the least self-descriptive.

**Table 2:** Summary of forced-choice models.

Response Format	Dominance Models	Unfolding Models
PICK	TIRT (Brown & Maydeu-Olivares, 2011), RIM (W.-C. Wang et al., 2017), MUPP-2PL (Hontangas et al., 2015), BRB-IRT (Lee & Smith, 2020), FC-DCM (Nájera et al., 2025), MGPPM (Furr & Fu, 2025)	MUPP-GGUM (Stark et al., 2005), ZG-MUPP (Joo et al., 2023)
RANK	TIRT, BRB-IRT, GLIRT (W.-C. Wang et al., 2016), ELIRT (W.-C. Wang et al., 2016), 2PLM-RANK (Zheng et al., 2024), MGPPM	GGUM-RANK (Joo et al., 2018)
MOLE	TIRT, BRB-IRT, GLIRT, ELIRT, MGPPM	GGUM-RANK

**Table 3:** Dataset summary.

	BFI-P	MAP	BFI-R	Sim-Mole
Number of Dimension	10	24	10	24
Number of Participants	499	1433	372	1000
Number of Items	50	264	60	480
Number of Item blocks	25	88	20	120
Items per item block	2	3	3	4
Item block type	PICK	RANK	RANK	MOLE

**Table 4:** Experimental results of participant performance prediction.

	BFI-P	MAP		BFI-R		Sim-Mole	
	**PRA**	**PRA**	**LRA**	**PRA**	**LRA**	**PRA**	**LRA**
Random	$0.502_{\pm 0.009}$	$0.500_{\pm 0.002}$	$0.166_{\pm 0.002}$	$0.498_{\pm 0.011}$	$0.164_{\pm 0.010}$	$0.500_{\pm 0.002}$	$0.083_{\pm 0.001}$
MUPP-2PL	$0.575_{\pm 0.013}$	$0.560_{\pm 0.004}$	$0.249_{\pm 0.004}$	$0.605_{\pm 0.025}$	$0.271_{\pm 0.024}$	$0.748_{\pm 0.001}$	$0.319_{\pm 0.000}$
BRB-IRT	$0.573_{\pm 0.013}$	$0.567_{\pm 0.003}$	$0.253_{\pm 0.003}$	$0.607_{\pm 0.020}$	$0.279_{\pm 0.022}$	$0.748_{\pm 0.001}$	$0.316_{\pm 0.002}$
MF	$0.650_{\pm 0.003}$	$0.573_{\pm 0.003}$	$0.264_{\pm 0.007}$	$0.649_{\pm 0.003}$	$0.311_{\pm 0.008}$	$0.689_{\pm 0.001}$	$0.243_{\pm 0.002}$
RankNet	$0.648_{\pm 0.002}$	$0.577_{\pm 0.003}$	$0.265_{\pm 0.003}$	$0.651_{\pm 0.007}$	$0.317_{\pm 0.009}$	$0.686_{\pm 0.002}$	$0.238_{\pm 0.002}$
NCDM-R	${\underline{0.671}}_{\pm 0.003}^{‡}$	${\underline{0.596}}_{\pm 0.002}^{‡}$	$0.270_{\pm 0.001}$	$0.681_{\pm 0.003}^{‡}$	$0.349_{\pm 0.005}$	${\underline{0.749}}_{\pm 0.001}$	${\underline{0.319}}_{\pm 0.001}$
KaNCD-R	$0.661_{\pm 0.005}$	$0.595_{\pm 0.003}^{‡}$	$0.268_{\pm 0.005}$	${\underline{0.681}}_{\pm 0.006}^{‡}$	${\underline{0.352}}_{\pm 0.008}$	$0.739_{\pm 0.001}$	$0.308_{\pm 0.001}$
CDND-R	$0.656_{\pm 0.006}$	$0.583_{\pm 0.002}$	${\underline{0.274}}_{\pm 0.003}$	$0.669_{\pm 0.005}$	$0.339_{\pm 0.004}$	$0.699_{\pm 0.002}$	$0.250_{\pm 0.001}$
FCNLT	$0.673_{\pm 0.003}$	$0.598_{\pm 0.001}$	$0.279_{\pm 0.002}^{†}$	$0.679_{\pm 0.004}$	$0.369_{\pm 0.007}^{†}$	$0.772_{\pm 0.001}^{†}$	$0.357_{\pm 0.001}^{†}$

Note. Values are ${\mathrm{Mean}}_{\pm \mathrm{SD}}$ over 10 independent runs. ^†^ Significantly better than all baseline models (Wilcoxon signed-rank test with Holm–Bonferroni correction, adjusted p<0.05). ^‡^ Not significantly different from FCNLT (adjusted p≥0.05); all other baseline values are significantly lower than FCNLT. Best results are **boldfaced**, and second-best are underlined.

**Table 5:** Computational efficiency comparison across models and datasets.

Model	Parameters	GPU (MB)	Epoch Time (s)	Best Epoch	Time to Best (s)
**BFI-P**					
MUPP-2PL	5090	0.14	$0.352_{\pm 0.005}$	$23.7_{\pm 18.111}$	$8.345_{\pm 6.337}$
BRB-IRT	17,565	0.42	$0.369_{\pm 0.006}$	$46.5_{\pm 35.041}$	$17.068_{\pm 12.748}$
MF	101,185	19.87	$0.812_{\pm 0.075}$	$3.5_{\pm 2.014}$	$2.708_{\pm 1.334}$
RankNet	101,185	19.9	$0.713_{\pm 0.066}$	$2.6_{\pm 1.350}$	$1.774_{\pm 0.791}$
NCDM-R	41,381	19.82	$0.535_{\pm 0.003}$	$92.1_{\pm 27.286}$	$49.288_{\pm 14.775}$
KaNCD-R	47,113	21.28	$0.743_{\pm 0.240}$	$112.0_{\pm 20.320}$	$82.079_{\pm 24.847}$
CDND-R	11,931	18.92	$0.652_{\pm 0.022}$	$52.0_{\pm 30.772}$	$33.943_{\pm 20.656}$
FCNLT	408,705	25.71	$3.508_{\pm 0.035}$	$1.1_{\pm 0.316}$	$3.851_{\pm 1.061}$
**MAP**					
MUPP-2PL	34,920	0.83	$10.872_{\pm 1.173}$	$71.0_{\pm 23.617}$	$770.508_{\pm 259.614}$
BRB-IRT	161,024	3.72	$11.446_{\pm 0.121}$	$75.8_{\pm 23.413}$	$868.951_{\pm 272.649}$
MF	174,657	21.5	$7.763_{\pm 0.101}$	$2.8_{\pm 1.549}$	$21.669_{\pm 11.975}$
RankNet	174,657	21.03	$10.890_{\pm 0.117}$	$5.5_{\pm 3.923}$	$59.676_{\pm 42.001}$
NCDM-R	80,417	20.67	$15.682_{\pm 0.191}$	$11.9_{\pm 2.514}$	$186.571_{\pm 39.372}$
KaNCD-R	74,151	23.86	$19.184_{\pm 3.280}$	$13.0_{\pm 3.266}$	$249.729_{\pm 73.519}$
CDND-R	86,761	27.47	$18.881_{\pm 2.530}$	$4.2_{\pm 1.229}$	$78.827_{\pm 22.624}$
FCNLT	2,317,825	69.39	$8.525_{\pm 0.264}$	$1.0_{\pm 0.000}$	$8.525_{\pm 0.264}$
**BFI-R**					
MUPP-2PL	3840	0.12	$0.618_{\pm 0.011}$	$85.1_{\pm 16.934}$	$52.604_{\pm 10.342}$
BRB-IRT	11,280	0.28	$0.658_{\pm 0.014}$	$85.4_{\pm 16.568}$	$56.207_{\pm 10.883}$
MF	93,697	20.26	$0.741_{\pm 0.054}$	$3.2_{\pm 1.317}$	$2.309_{\pm 0.832}$
RankNet	93,697	19.79	$0.895_{\pm 0.120}$	$5.4_{\pm 4.575}$	$4.346_{\pm 3.416}$
NCDM-R	40,221	19.81	$0.928_{\pm 0.011}$	$71.7_{\pm 22.706}$	$66.544_{\pm 21.260}$
KaNCD-R	44,783	21.26	$1.038_{\pm 0.008}$	$59.7_{\pm 11.324}$	$61.929_{\pm 11.747}$
CDND-R	9591	18.89	$0.930_{\pm 0.006}$	$36.2_{\pm 29.427}$	$33.640_{\pm 27.247}$
FCNLT	328,705	26.3	$1.400_{\pm 0.639}$	$8.1_{\pm 3.604}$	$11.583_{\pm 8.135}$
**sim-mole**					
MUPP-2PL	24,960	0.6	$18.656_{\pm 2.921}$	$80.0_{\pm 12.841}$	$1474.467_{\pm 230.680}$
BRB-IRT	144,960	3.35	$18.568_{\pm 1.080}$	$86.2_{\pm 11.708}$	$1602.391_{\pm 248.042}$
MF	160,769	21.79	$9.835_{\pm 0.081}$	$6.9_{\pm 2.470}$	$67.804_{\pm 24.136}$
RankNet	160,769	20.81	$17.085_{\pm 0.178}$	$4.2_{\pm 3.490}$	$71.491_{\pm 58.701}$
NCDM-R	75,425	20.59	$26.399_{\pm 3.030}$	$5.8_{\pm 0.633}$	$153.709_{\pm 28.317}$
KaNCD-R	70,027	23.8	$28.883_{\pm 1.572}$	$21.1_{\pm 13.699}$	$615.555_{\pm 408.816}$
CDND-R	76,345	27.31	$30.788_{\pm 4.331}$	$16.6_{\pm 6.168}$	$503.188_{\pm 191.184}$
FCNLT	1,680,385	55.04	$33.869_{\pm 1.711}$	$4.3_{\pm 0.675}$	$145.189_{\pm 20.999}$

Note. Epoch Time, Best Epoch, and Time to Best are reported as ${\mathrm{Mean}}_{\pm \mathrm{SD}}$ over five independent runs. Time to Best is computed as (total training time/number of trained epochs) × best epoch, eliminating the influence of different stopping criteria.

**Table 6:** Ablation experiments.

	BFI-P	MAP		BFI-R		sim-mole	
	**PRA**	**PRA**	**LRA**	**PRA**	**LRA**	**PRA**	**LRA**
FCNLT_EB	$0.674_{\pm 0.005}$	$0.597_{\pm 0.004}$	$0.277_{\pm 0.002}$ *	$0.678_{\pm 0.003}$	$0.356_{\pm 0.006}$ *	$0.771_{\pm 0.001}$	$0.357_{\pm 0.001}$
FCNLT_BPR	$0.665_{\pm 0.005}$	$0.594_{\pm 0.002}$ *	$0.261_{\pm 0.003}$ *	$0.672_{\pm 0.003}$ *	$0.332_{\pm 0.005}$ *	$0.742_{\pm 0.001}$ *	$0.307_{\pm 0.001}$ *
FCNLT_List	$0.665_{\pm 0.005}$	$0.584_{\pm 0.002}$ *	$0.272_{\pm 0.005}$ *	$0.669_{\pm 0.003}$ *	$0.332_{\pm 0.011}$ *	$0.726_{\pm 0.001}$ *	$0.284_{\pm 0.001}$ *
FCNLT_MO	$0.668_{\pm 0.006}$	${\underline{0.598}}_{\pm 0.002}$	${\underline{0.278}}_{\pm 0.004}$	$0.679_{\pm 0.003}$	${\underline{0.368}}_{\pm 0.006}$	${\underline{0.772}}_{\pm 0.001}$	$0.359_{\pm 0.002}$
FCNLT	${\underline{0.673}}_{\pm 0.003}$	$0.598_{\pm 0.001}$	$0.279_{\pm 0.002}$	${\underline{0.679}}_{\pm 0.004}$	$0.369_{\pm 0.007}$	$0.772_{\pm 0.001}$	${\underline{0.357}}_{\pm 0.001}$

Note. Values are ${\mathrm{Mean}}_{\pm \mathrm{SD}}$ over 10 independent runs. * Significantly worse than the full FCNLT model (Wilcoxon signed-rank test with Holm–Bonferroni correction, adjusted p<0.05). Best results are **boldfaced**, and second-best are underlined.

**Table 7:** Parameter recovery results on the sim-mole dataset.

Parameter	Pearson *r*	Min	Max
Latent trait (24 dims)	$0.639_{\pm 0.051}$	0.505	0.738
Item discrimination	$0.871_{\pm 0.014}$	0.837	0.884
Item difficulty	$0.559_{\pm 0.007}$	0.548	0.572

Note. Values are reported as mean ± standard deviation over 10 independent runs, along with the minimum and maximum dimension- or item-level mean correlations. All correlations are significant at p<0.001.

## Data Availability

The raw data supporting the conclusions of this article will be made available by the authors on request.

## Note

1 In classical psychometric theory, IRT and CDMs are methodologically distinct, focusing on continuous latent traits and discrete attribute mastery, respectively. However, recent deep learning-based approaches often do not strictly maintain this distinction in their architectures. Therefore, in this paper, when referring to deep learning-based methods, we retain the term “cognitive diagnostic models”; when describing the model proposed in this study, we use the more neutral term “latent trait model” to avoid conceptual confusion.

## Appendix A

### Appendix A.1. Comparison of NCDM-R and Its BPR Loss Variant

To further verify the validity of adopting the RankNet pairwise probability formula as the unified baseline adaptation strategy, we modified the best-performing baseline NCDM-R by replacing its loss function with the BPR loss (denoted as NCDM-bpr). The predictive performance of the two models on the four datasets is shown in Table A1. Paired Wilcoxon signed-rank tests with Holm–Bonferroni correction showed no significant differences between the two versions across all datasets and metrics (adjusted p>0.05). This result indicates that applying the BPR loss to the baseline model does not yield a consistent advantage over the RankNet formula, thereby justifying the rationale of employing the RankNet probability layer as a unified and minimal adaptation strategy for all neural baselines in this study.

**Table A1:** Experimental results of NCDM-R and NCDM-bpr for participant performance prediction.

	BFI-P	MAP		BFI-R		sim-mole	
	**PRA**	**PRA**	**LRA**	**PRA**	**LRA**	**PRA**	**LRA**
NCDM-R	$0.671_{\pm 0.003}$	$0.596_{\pm 0.002}$	$0.270_{\pm 0.001}$	$0.681_{\pm 0.003}$	$0.349_{\pm 0.005}$	$0.749_{\pm 0.001}$	$0.319_{\pm 0.001}$
NCDM-bpr	$0.669_{\pm 0.004}$	$0.597_{\pm 0.002}$	$0.270_{\pm 0.001}$	$0.680_{\pm 0.002}$	$0.353_{\pm 0.005}$	$0.749_{\pm 0.001}$	$0.319_{\pm 0.001}$

Note. Values are ${\mathrm{Mean}}_{\pm \mathrm{SD}}$ over 10 independent runs.

### Appendix A.2. 5-Fold Cross-Validation of FCNLT on the BFI Datasets

To verify the robustness of the evaluation results on the BFI datasets, which have relatively small sample sizes, this study conducted a 5-fold cross-validation on BFI-P and BFI-R, respectively, with 10 independent random seeds run for each fold. The means and standard deviations of PRA and LRA for each fold, the cross-validation mean, and the 80/20 split results reported in the original Table 4 are summarized in Table A2. Paired Wilcoxon signed-rank tests were conducted to compare the results of each fold with the original split, as well as pairwise among the five folds, using the Holm–Bonferroni correction to control the family-wise error rate for multiple comparisons.

For BFI-P, comparing each fold with the results in the original Table 4, the adjusted tests showed significant differences in PRA between the original split and Folds 2, 3, and 4, whereas Folds 1 and 5 showed no significant differences from the original split. In the pairwise comparisons among the five folds, the specific combinations exhibiting significant differences were: Fold 1 vs. Folds 2, 3, and 5; Fold 2 vs. Fold 4; Fold 3 vs. Fold 4; and Fold 4 vs. Fold 5. The remaining pairwise comparisons among the folds showed no significant differences. The likely reason for the higher number of significant differences observed on BFI-P is that this dataset uses the PICK-2 format. Under the 80/20 split, the test set contains 5 item blocks for each of the 499 participants, totaling 2495 valid data points. For deep learning models, this data volume is relatively small, leading to larger performance fluctuations across folds and making it easier for statistical tests to detect differences.

For BFI-R, comparing each fold with the results in the original Table 4, the adjusted tests indicated that only Fold 4 exhibited a significant difference in PRA from the original split. Furthermore, this difference was in a positive direction (0.689>0.679), suggesting that the original split did not overestimate the model’s performance. The remaining folds showed no significant differences from the original split. In all pairwise comparisons among the five folds, neither PRA nor LRA showed any significant differences. The high stability of the cross-validation results on BFI-R can be explained from a data volume perspective: although BFI-R has fewer participants (372) than BFI-P (499), it adopts the RANK-3 format. Under the 80/20 split, each participant contributes 4 item blocks to the test set, and each block contains 3 valid pairwise comparisons, resulting in a total of 372×4×3=4464 valid data points in the test set, which is approximately 1.8 times that of BFI-P. This more abundant volume of valid data provides a stronger statistical foundation for model evaluation, thereby leading to more consistent performance across the folds.

**Table A2:** 5-fold cross-validation results of FCNLT on the BFI datasets.

	BFI-P	BFI-R	
	**PRA**	**PRA**	**LRA**
Fold 1	$0.674_{\pm 0.006}$	$0.684_{\pm 0.003}$	$0.366_{\pm 0.006}$
Fold 2	$0.658_{\pm 0.005}$	$0.680_{\pm 0.006}$	$0.364_{\pm 0.006}$
Fold 3	$0.661_{\pm 0.006}$	$0.684_{\pm 0.006}$	$0.367_{\pm 0.007}$
Fold 4	$0.676_{\pm 0.004}$	$0.689_{\pm 0.005}$	$0.368_{\pm 0.007}$
Fold 5	$0.659_{\pm 0.004}$	$0.682_{\pm 0.007}$	$0.362_{\pm 0.007}$
CV Mean	$0.665_{\pm 0.009}$	$0.684_{\pm 0.006}$	$0.365_{\pm 0.007}$
Original (Table 4)	$0.673_{\pm 0.003}$	$0.679_{\pm 0.004}$	$0.369_{\pm 0.007}$

Note. Values are reported as ${\mathrm{Mean}}_{\pm \mathrm{SD}}$ over 10 independent runs per fold. “CV Mean” denotes the average across the five folds. “Original” refers to the single 80/20 split reported in Table 4.
